# Clinical application of external fixation in preventing displacement of high-position airway stents: 2 case reports

**DOI:** 10.1097/MD.0000000000045839

**Published:** 2025-11-07

**Authors:** Luna Yuan, Yun Liu, Yipeng Li, Jinhua Zhou, Liran Zhang, Wen Zhang, Shanshan Zhang, Cuixia Bian

**Affiliations:** a Department of Pulmonary and Critical Care Medicine, Jining No. 1 People’s Hospital, Jining, Shandong Province, P.R. China; b Clinical Medical College of Jining Medical University, Jining Medical University, Jining, Shandong Province, P.R. China; c Department of Pulmonary and Critical Care Medicine, Qufu People’s Hospital, Jining, Shandong Province, P.R. China.

**Keywords:** external stabilization technique, high-position airway stenosis, stent migration, tracheal stent, tracheoesophageal fistula

## Abstract

**Rationale:**

Airway stenosis and tracheoesophageal fistulas are life-threatening emergencies, often complicated by asphyxia, recurrent infections, and fatal outcomes. Tracheal stent implantation is effective, but stent migration remains a critical complication, especially in subglottic high-position airway pathologies. This report aims to demonstrate an external stabilization technique to prevent stent migration in this high-risk group. Such cases are rarely reported.

**Patient concerns:**

Two representative clinical cases with subglottic high-position airway pathologies requiring stent implantation.

**Diagnoses:**

Tracheoesophageal fistula, high-position airway stenosis.

**Interventions:**

The methods employed were either percutaneous suture fixation or snare wire anchorage, designed to achieve reliable stent immobilization. A modified snare wire fixation protocol was specifically developed to address the limitation of suture material failure.

**Outcomes:**

After repeated follow-up, the stents in both patients did not shift.

**Lessons:**

The innovative utilization of either percutaneous suture fixation or snare wire anchorage achieved reliable stent immobilization. The modified snare wire fixation technique offers enhanced long-term stability by overcoming the inherent risk of suture material failure and maintained an optimal safety profile and cost-effectiveness.

## 1. Introduction

For airway stenosis or airway fistulas up to 2 cm below the glottis, straight-tube airway stents are often selected because of the relatively high lesion location.^[1]^ However, considering the significant mobility of the neck and the gradual increase in the tracheal diameter from the upper to the lower part, stents are highly prone to displacement after implantation. Migrated stents not only fail to achieve therapeutic objectives but may also critically compromise airway patency, potentially culminating in life-threatening asphyxia. Here, we present a novel approach involving external fixation of tracheal stents to mitigate migration risks. Our cases suggest that this external fixation method is a safe, cost-effective, and clinically viable solution for enhancing stent stability during upper airway interventions.

## 2. Case presentation

### 2.1. Case 1

**Figure 1.**
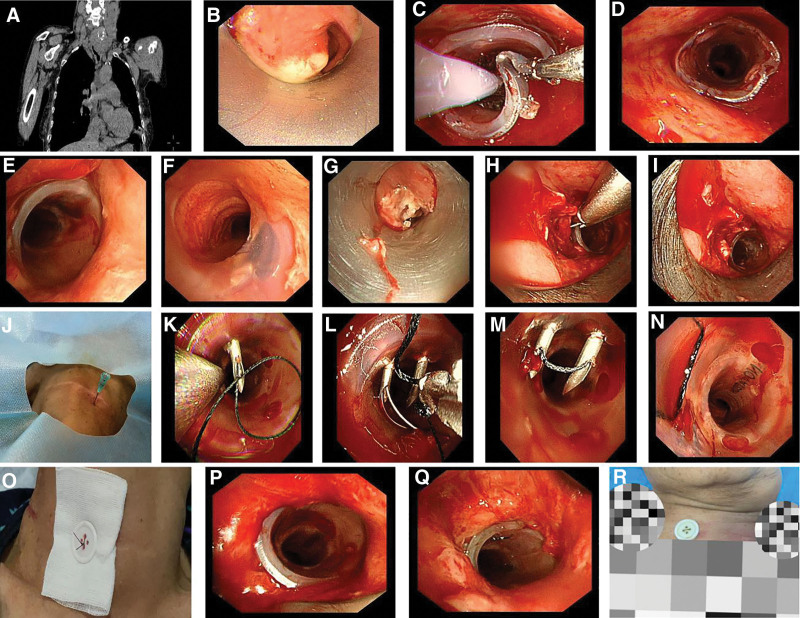
(A) Pre-operative chest CT findings: thyroid mass with extrinsic compression-induced stenosis of the large airway below the glottis. (B) Bronchoscopic findings: extrinsic and endophytic stenosis of the subglottic airway. (C) Balloon dilation for stent shaping after silicone stent implantation assisted by a rigid bronchoscope. (D) The stent fits well, and the lumen is patent after implantation. (E) Upper edge of the silicone stent. (F) Lower edge of the silicone stent. (G) A neoplasm was visible in the lumen below the glottis, causing lumen stenosis. (H) After removing the neoplasm, the silicone stent was found to have migrated downward. The upper edge of the stent was clamped and rotated upward for repositioning. (I) After adjustment, the stent was in a good position and the lumen was patent. (J) Determination of the tracheal compression site. A 20 mL syringe needle was inserted along the puncture site. (K) A suture was threaded along the puncture needle. (L) The deep-vein catheterization needle was inserted again. The suture was guided into the snare along with the needle and then clamped by a biopsy forceps and fully inserted into the snare. (M) The puncture needle brought the suture out of the body surface. (N) The suture fixed the silicone stent. (O) Fixing the suture by knotting with a button outside the body surface. (P) Patency of the upper airway after the placement and fixation of the silicone stent. (Q) The silicone stent was in an optimal position, and the lumen remained unobstructed. (R) The skin surrounding the external fixation exhibited no signs of erythema or swelling.

A 78-year-old female presented with a 4-day history of progressive dyspnea requiring urgent evaluation. Her medical history was significant for thyroid carcinoma 17 years after the total thyroidectomy. She reported rapidly worsening respiratory distress accompanied by a productive cough during the 4-day prodromal period. Initial diagnostic imaging revealed critical central airway stenosis secondary to suspected recurrent thyroid carcinoma with tracheal invasion (Fig. 1A). On October 5, 2021, rigid bronchoscopy demonstrated critical subglottic stenosis with 95% luminal obstruction extending over a 50-mm segment (Fig. 1B). The stenotic region proved impassable to standard bronchoscopic instrumentation, necessitating precise debridement of the obstructing granulation tissue followed by sequential balloon dilation. Post-interventional airway evaluation revealed intact mucosal integrity in the mid-to-distal trachea with preserved luminal patency and well-defined carinal anatomy. A customized 16-14-16 mm hourglass-shaped silicone stent was selected based on 3-dimensional tracheal measurements. Prophylactic anti-migration modifications were implemented through strategic stent fenestration before deployment. Immediate postprocedural assessment confirmed optimal stent apposition and complete airway continuity restoration (Fig. 1C and D). Surveillance bronchoscopy on October 6, 2021, demonstrated maintained stent positional integrity without migration evidence (Fig. 1E and F). The patient achieved significant symptomatic resolution and was discharged with stable respiratory function. On October 13, 2021, the patient developed acute-onset dyspnea and tachypnea without identifiable triggers, requiring immediate bronchoscopic evaluation. Endoscopic findings revealed recurrent neoplastic proliferation in the subglottic region with luminal compromise (Fig. 1G). After excision of the obstructive granulation tissue, stent migration was confirmed by caudal displacement of the hourglass-shaped silicone prosthesis. Successful endoscopic repositioning was achieved through rotational traction applied to the proximal stent margin, restoring anatomical alignment and airway patency (Fig. 1H and I). The patient presented with recrudescent respiratory distress on October 24, 2021. Urgent bronchoscopy revealed recurrent stent malpositioning and established a pattern of prosthetic instability. Given this cumulative evidence of the migration propensity, a decision was made to implement external suture fixation as a secondary stabilization strategy.

Specific operation method: the location of tracheal stenosis was determined based on computed tomography 3-dimensional reconstruction. A puncture needle A (the needle of a 20 mL syringe) was inserted along the positioned site (Fig. 1J). Then, a suture was threaded along the puncture needle A (Fig. 1K). After that, a puncture needle B (the needle of a 20 mL syringe) was inserted again at an interval of 5 mm. A snare was introduced along the puncture needle B, and the suture was clamped by a biopsy forceps and sent into the snare (Fig. 1L). The snare was retrieved, and the suture was pulled out of the body surface (Fig. 1M and N). The suture was fixed and knotted with a button outside the body surface (Fig. 1O). The stent was then placed in an appropriate position (Fig. 1P). On October 26, a bronchoscopic reexamination was performed (Fig. 1Q), and the silicone stent was found to be in a good position without displacement. Multiple subsequent hospitalizations for bronchoscopic reexamination showed no stent displacement, and there was no bleeding or infection on the skin surface of the external fixation (Fig. 1R).

### 2.2. Case 2

**Figure 2.**
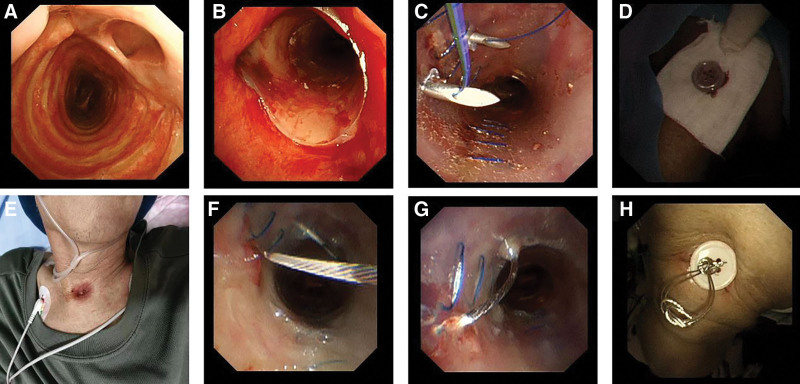
(A) On July 4, 2024, a longitudinal tracheoesophageal fistula approximately 10 mm in length was visualized 20 mm below the glottis. (B) Implantation of the modified stent. (C) External fixation of the stent with sutures from the inside. (D) External fixation of the sutures on the body surface. (E) Rupture of the external fixation suture on the body surface. (F and G) External fixation with snare wire instead of suture. (H) The body surface after external fixation with a snare wire.

A 58-year-old male patient was transferred to our hospital on June 25, 2024, due to tracheoesophageal fistula. Bronchoscopy was performed on July 4, 2024. A longitudinal tracheoesophageal fistula, approximately 10 mm in length, was visible 20 mm below the glottis (Fig. 2A). Local luminal stenosis was observed at the upper edge of the fistula orifice. A modified, straight-tube-shaped silicone stent with an enlarged diameter was implanted. After implantation, the stent was placed to cover the fistula orifice (Fig. 2B). On July 7, 2024, a reexamination bronchoscope revealed the stent had shifted 5 mm distally, exposing the fistula orifice edge, with digestive fluid leakage around it. Under a rigid bronchoscope, grasping forceps adjusted the stent’s position, and external fixation with sutures was performed (Fig. 2C and D). On July 10, 2024, the external fixation suture ruptured (Fig. 2E), and bronchoscopy showed the stent had shifted again. To prevent suture rupture, the fixation method was changed to external fixation using a snare wire (Fig. 2F and G). A reexamination bronchoscope on July 17 showed no further displacement, and no complications such as pain, bleeding, or infection were observed on the skin around the external fixation (Fig. 2H).

## 3. Discussion

Large airway stenosis or tracheoesophageal fistula are critical, life-threatening conditions. In severe cases, they can cause infection, asphyxia, and death. Recently, clinical application of endobronchial stents has achieved remarkable therapeutic effects.^[2]^ However, after implantation, especially in high-position airway lesions below the glottis, stent displacement often occurs, seriously affecting treatment outcomes.

The causes of high-positioned airway stent displacement relate to factors like stenosis location, lesion nature, stent model selection, and neck mobility.^[3]^ Both patients had high-position airway lesions. After assessment, silicone stents were chosen. However, due to the lesions’ high location, large trachea diameter, extrinsic compression-induced stenosis, and extensive neck movement, the stents repeatedly displaced after implantation.

To reduce displacement, accurate measurements are crucial for selecting suitable stents. For high-position airway stenosis near the glottis, the stent should not be too large to minimize glottic and laryngeal irritation. Holes created at the stent’s lower end allow granulation tissue hyperplasia for fixation, but this requires time and may block the lumen. Standardized atomized inhalation and cough-suppressing treatments were performed post-implantation to reduce airway reactions and stent movements. Despite these measures, the stent still displaced repeatedly, necessitating a more feasible fixation method.

A study^[4]^ showed that esophageal stents with hanging sutures can reduce displacement. Pre-setting retrieval lines at the upper opening of covered stents and fixing them at lesion sites effectively reduces displacement. Various stent fixation techniques have been developed, but none widely popularized. Andreetti^[5]^ reported an external fixation method for upper airway silicone stents using absorbable sutures. Currently, external fixation techniques for preventing tracheal stent displacement are rare.

At our center, both cases involved high-positioned airway lesions with stents displacing shortly after implantation. In Case 1, after the stent shifted, external fixation with sutures was applied, and no further displacement occurred. In Case 2, the high lesion location and significant local muscle tension, resulting in the rupture of the external fixation sutures. The innovative use of a snare wire for external fixation prevented suture failure, thereby preventing further stent displacement. External fixation with sutures or snare wires does not increase complications of tracheal stent implantation. No stent damage, dislocation, blockage, or vocal cord dysfunction was observed. Post-operation, careful disinfection management of the skin around the fixation site prevented complications like infection, bleeding or pain.

In summary, external fixation effectively prevents tracheal stent displacement and is clinically safe and effective. Despite the promising outcomes observed in these 2 cases, several limitations should be acknowledged. First, the current follow-up period remains relatively short, and there is a risk of fatigue fracture in sutures and snare wires, necessitating continued close monitoring. Second, the small sample size limits the generalizability of these findings, requiring larger-scale prospective studies to validate the efficacy and safety of this approach.

## Acknowledgments

We would like to thank Editage for English language editing.

## Author contributions

**Conceptualization:** Luna Yuan, Cuixia Bian.

**Data curation:** Wen Zhang, Shanshan Zhang.

**Methodology:** Cuixia Bian.

**Supervision:** Cuixia Bian.

**Writing – original draft:** Luna Yuan, Yun Liu.

**Writing – review & editing:** Yipeng Li, Jinhua Zhou, Liran Zhang.
